# More Than Three Decades After Discovery of the Neuroprotective Effect of PACAP, What Is Still Preventing Its Clinical Use?

**DOI:** 10.1007/s12031-025-02366-z

**Published:** 2025-06-21

**Authors:** Asma Cherait, Xavier Xifró, Dora Reglodi, David Vaudry

**Affiliations:** 1 Department of Second Cycle, Higher School of Agronomy Mostaganem, 27000 Mostaganem, Algeria; 2 Laboratory of Cellular Toxicology, University of Badji Mokhtar, 23000 Annaba, Algeria; 3 Inserm U1245, Univ Rouen Normandie, Normandie Univ, 76000 Rouen, France; 4 New Therapeutic Targets Group, Department of Medical Science, Faculty of Medicine, Universitat de Girona, 17003 Girona, Spain; 5 Department of Anatomy, HUN-REN-PTE PACAP Research Team, Centre for Neuroscience, Medical School, University of Pecs, 7624 Pecs, Hungary

**Keywords:** PACAP, Neuroprotection, Clinical use, Neurodegenerative diseases

## Abstract

Discovered in 1989, pituitary adenylate cyclase-activating polypeptide (PACAP) is a neuropeptide with strong neuroprotective properties, as shown in various neurodegenerative preclinical models of Parkinson, Alzheimer, or Huntington diseases. PACAP neuroprotection has also been reported in animal models of cerebral ischemia and traumatic brain injury. The neuroprotective effect of PACAP occurs through its capacity to modulate most of the multiphasic aspects of neuronal diseases, such as oxidative stress, neuronal cell death, and inflammation. However, more than three decades after its discovery, and although PACAP neurotrophic and neuroprotective activities have now been largely documented, its clinical use is still awaited. Thus, the aim of this manuscript is to discuss the main reasons which limit the use of PACAP as a therapeutic agent for the treatment of neuronal diseases. To achieve this objective, an opinion survey has been conducted among experts in the field of PACAP, and a bibliographic investigation was carried out.

**Supplementary Information:**

The online version contains supplementary material available at 10.1007/s12031-025-02366-z.

## Introduction

Neurological disorders are the world’s leading cause of disability, affecting more victims every year, and the cost of medical care for these dependent individuals continues to increase without effective treatment being offered. Therefore, emphasis is on therapeutic approaches that could address this unmet clinical need. In this context, pituitary adenylate cyclase-activating polypeptide (PACAP), a very conserved 38 amino acid polypeptide identified by the team of Professor Arimura from hypothalamic extracts for its ability to stimulate adenylate cyclase (Miyata et al. 1989; Miyata et al. 1990; Arimura 2007), seems to be a promising therapeutic agent to treat both acute brain insults and also chronic neurodegenerative disorders (Lee and Seo 2014). Indeed, in rodents, PACAP reduces stroke infarct volume and promotes functional recovery after transient and permanent middle cerebral artery occlusion (MCAO) (Banks 1996; Reglodi 2000; Cherait et al. 2021). It also exerts strong neuroprotective effects in mice subjected to traumatic brain injury (TBI) (Miyamoto et al. 2014; Shioda and Nakamachi 2015), prevents Alzheimer and Parkinson’s diseases in both in vitro and in vivo models (Rat et al. 2011; Lamine et al. 2016; Maasz et al. 2017; Schaler et al. 2021; Wang et al. 2023), protects from neuronal death, and improves memory performance in a Huntington’s disease model (Cabezas-Llobet et al. 2018; Solés-Tarrés et al. 2022a, b). An extraordinary feature of PACAP is its ability to counteract most of the numerous deleterious mechanisms activated in various neuronal diseases through a blockage of excitotoxicity, a reduction of oxidative stress, a modulation of the inflammatory response, and an inhibition of apoptosis (Djeda and al. 2011; Cherait et al. 2021). Furthermore, PACAP promotes neuronal plasticity (Cabezas-Llobet et al. 2018; Shibato et al. 2023) which contributes to functional recovery.

**Fig. 1:**
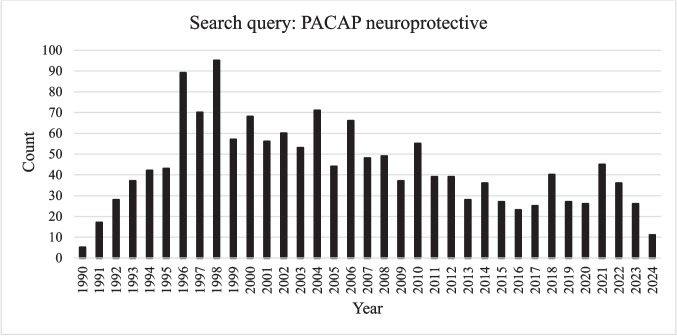
Number of studies addressing the PACAP neuroprotective effects from 1990 to December 2024 in PubMed

These examples only represent a small illustration of the numerous studies regarding the neurotrophic and neuroprotective effects of PACAP after brain injury, which have been reported over the last three decades. A PubMed search from January 1990 to December 2024 on PACAP reports over 4500 studies, while narrowing the search to “PACAP neuroprotective” results in 1413 studies (Fig. 1) which is an average of 40 publications/year on this topic and represents more than 30% of all PACAP-related publications. Search on “PACAP protective” results in only a little higher number of publications (1680), showing that the protective effects of PACAP are not limited to neuroprotection. Although PACAP has general cytoprotective effects, its neuroprotective activity has drawn major attention. Research on this topic has been particularly intense from 1996 to 2006, with an average of 66 publications by year. Professor Akira Arimura, who discovered PACAP and inspired a whole generation of researchers, also filed a patent in 1997 entitled “Method and pharmaceutical composition for prevention and treatment of brain damage” (Patent number: 6680295). After this prolific period, the research on PACAP neuroprotective effects stepped down over the last decade. This could be due to the fact that, since its discovery, sufficient results have been accumulated to demonstrate the neuroprotective effect of PACAP in various preclinical models to now enable clinical studies. However, very few human clinical trials have been undertaken so far, which may have diverted researchers towards other molecules with more immediate therapeutic applications.

This manuscript tries to explain what hinders the use of PACAP in clinic and proposes some solutions that could contribute to overcoming the encountered problems in order to take advantage of this promising therapeutic candidate for the treatment of neuronal diseases.

## Methodology

A systematic PubMed search has been conducted to revise the literature from 1990 to December 2024.

In addition to that, a survey has been carried out among researchers who have published more than two publications in a journal referenced in PubMed regarding the neuroprotective effect of PACAP. Within the hundred of specialists solicited, 46 responded to our survey, and we thank them for that. The survey included 20 questions as shown in the appendix. The most relevant question of this poll for this review was “Despite the powerful neuroprotective effects that have been reported for more than 20 years now, why do you think PACAP is still not used in clinic for treatment of cerebral ischemia or some neurodegenerative diseases?” This questionnaire aimed to figure out where we are regarding the clinical use of PACAP and what is still preventing its use for the treatment of cerebral ischemia or some neurodegenerative diseases, and we have encouraged the participants to mention some references which support their answers/opinions.

Our methodology was based on the responses obtained from the specialists and the revision of the literature research.

## Results and Discussion

**Fig. 2:**
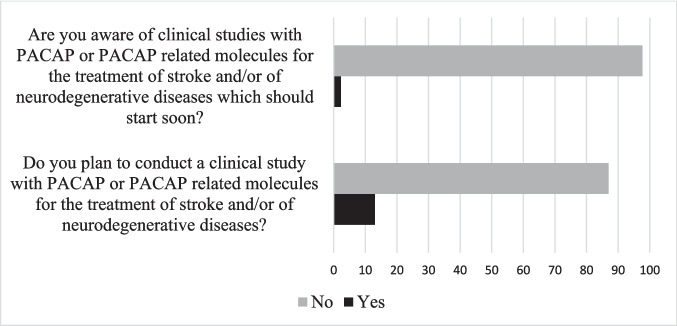
Specialists’ response by percentage regarding potential clinical studies with PACAP

Despite all the reported neuroprotective effects in preclinical studies, the fact that only 2% of the specialists in the field were aware of clinical studies with PACAP or PACAP-related molecules for the treatment of stroke and/or of neurodegenerative diseases, which should start soon (Fig. 2), confirms the lack of interest among clinicians for this neuropeptide. Furthermore, only 12% of the survey respondents plan to conduct a trial in the future. Among the pathologies they plan to treat with PACAP or PACAP-related molecules, we can mention Parkinson’s disease, multiple sclerosis, Huntington’s disease, fetal alcohol syndrome, and diabetic retinopathy.

PACAP research has primarily remained at the preclinical stage for neuroprotection applications, and although the evidence demonstrates PACAP’s biological activity and potential therapeutic applications, the barriers to further therapeutic development stem largely from pharmacokinetic limitations, delivery challenges, and potential side effects. However, initial human pharmacokinetics (Birk et al. 2007) and clinical studies for another therapeutic application (Li and al. 2007; Doberer et al. 2007) took place 18 years ago with no serious side effects. Most were early-phase clinical trials with limited patient numbers, which highlighted the challenges that would need to be overcome for larger-scale development. Furthermore, much of this research have focused primarily on migraine provocation studies that were designed to understand PACAP’s role in triggering migraine attacks rather than harnessing its therapeutic potential (ClinicalTrials.gov ID ׃ NCT02158221, NCT02364453, NCT03471039, NCT03560024, NCT03881644). The absence of published clinical trials specifically evaluating PACAP as a neuroprotective agent in neurological conditions represents a significant translational gap that warrants closer examination.

This manuscript aims to explore the reasons behind clinicians’ limited focus on PACAP as a therapeutic agent, despite its demonstrated neuroprotective effects in countless preclinical studies. Based on the responses to the questionnaire and the bibliographic analysis, various obstacles to the use of PACAP in clinic for the treatment of neuronal diseases have been identified, ranging from the poor stability of the peptide in biological fluids, its numerous side effects, and its need to cross the blood–brain barrier, as discussed below.

### Blood-Brain Barrier (BBB) Crossing

The capacity of PACAP to bypass the blood–brain barrier (BBB), a very selective functional barrier that restricts the ability of numerous biomolecules to enter the brain, is an element meant to facilitate its use for the treatment of neuronal diseases. In normal conditions, PACAP38 (referred also as PACAP in this manuscript) can be transported actively by a saturable protein transport system-6 (PTS-6), localized in the endothelium. The shorter form of PACAP (PACAP27), which shares the same N-terminal amino acid sequence but has a shorter C-terminus (Arimura and Shioda 1995), is also able to cross the BBB to the brain (influx) by membrane diffusion, but after crossing the endothelial membrane, both isoforms are either rapidly degraded or efflux back from brain to blood by a saturable peptide carrier mechanism (Amin and Schytz 2018). However, after brain injury, a disruption of the BBB occurs, which could impact PACAP transport. In spinal cord injury, a decrease in PTS-6 activity has been observed after the lesion, but it is a transient drop with a return to normal activity after a week (Banks et al. 1998). Following cerebral ischemia, PACAP transport across the BBB was also dramatically reduced from 6 to 12 h after MCAO in all brain regions (Somogyvári-Vigh et al. 2000). Such transient alteration of PACAP transport was observed in a traumatic brain injury animal model too, but with some brain regional differences in PACAP uptake, since the loss of PACAP capacity to cross the BBB was lower in the cerebellum than in the cerebral cortex (Rhea et al. 2018). On the other hand, intraperitoneal injection of lipopolysaccharide did not alter the efflux of radioactively labeled PACAP from the central nervous system, even after BBB disruption. However, the radioactive PACAP influx was reduced, but PTS-6 activity remained sufficient to deliver enough peptide to the brain to exert a neuroprotection (Nonaka et al. 2005). Interestingly, PACAP and its receptors are expressed in the Virchow–Robin spaces (Staines et al. 2009) where they play an important role in maintaining the BBB’s functional integrity. This was shown in PACAP knock-out endothelial cells, where after subarachnoid hemorrhage, an exogenous administration of PACAP reinforces tight junctions of the BBB and attenuates brain edema (Fang et al. 2020). Therefore, depending on the disease, it appears that PACAP uptake could be more or less impacted. Consequently, to optimize the neuroprotective action of PACAP, it will be important to understand how each brain disorder affects PACAP influx and efflux in order to adjust the amount of PACAP to be administered. As proposed by some experts, PACAP analogs with a higher capacity to cross the BBB in a non-saturable and diffusible manner could also be helpful, such as PACAP glycopeptide analogs that have enhanced stability and BBB penetration, while they retained their neurotrophic and neuroprotective effects (Apostol et al. 2022; Bernard et al. 2024). Alternatively, the specialists reviewed have also proposed the use of nanocarriers. For instance, the use of liposomes functionalized with herpes virus-derived gH625 peptide sounds like a promising strategy to overcome the BBB obstacle and safely enhance PACAP delivery to the brain (Iachetta et al. 2019 and Barra et al. 2022a, b).

### Short Plasma Half-Life, Metabolic Instability, and Low Bioavailability

**Fig. 3:**
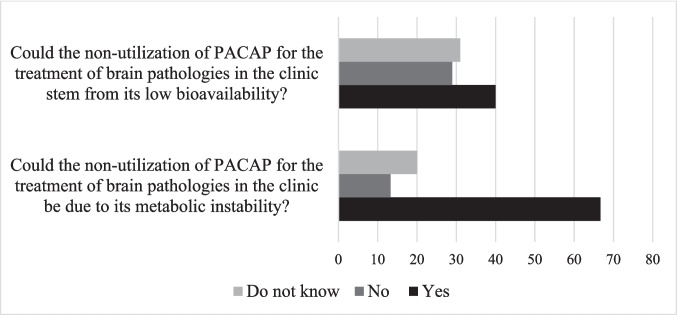
Response to the survey regarding the PACAP metabolic instability and low bioavailability

The metabolic instability and short plasma half-life of PACAP were cited by two-thirds of the researchers surveyed as limiting factors for its use in clinic to treat brain pathologies. In addition, 40% of the experts on PACAP consider that the low bioavailability of the peptide also impairs its clinical use (Fig. 3).

The expert’s response regarding PACAP metabolic instability is due to the fact that PACAP38, the dominant form in mammals’ tissue (Arimura et al. 1991; Arimura 1992), undergoes a rapid degradation by various peptidases, such as the dipeptidyl peptidase-IV (DPP-IV) and carboxypeptidases, with less than 5 min of half-life in vivo (Vaudry et al. 2000). This rapid cleavage of PACAP by DPP-IV produces PACAP5-38, which can act as a PAC1 antagonist (Bourgault et al. 2009a). PACAP27, which represents less than 10% of total PACAP, seems to be more resistant to enzymatic degradation than PACAP38 (Bourgault et al. 2008). But in the end, it is estimated that the quantity of intact PACAP that reaches the brain, under normal conditions in mice, is between% 0.12 and 0.15% of the intravenously (IV) injected peptide (Banks 2016; Rhea et al. 2018); the rest being rapidly eliminated.

Although the low bioavailability is considered a major obstacle, this should not necessarily be the main reason for disregarding the therapeutic use of PACAP. Comparing this short half-life with other peptides reveals that most peptide hormones have similarly short half-life and many of them are in clinical use. For example, the 14 amino acid somatostatin has a half-life of 1–3 min, cleaved by peptidases (Sheppard et al. 1979; Rai et al. 2015), the much bigger parathormon (84 amino acids) and calcitonin (32 amino acids) also have a half-life of a few minutes (Martin et al. 1998). Oxytocin, which is often used in obstetrics, is cleared in 3–6 min (Rydén and Sjöholm 1969; Fabian et al. 1969). Other routes of administration may overcome this difficulty (see below section PACAP delivery route). Another aspect of PACAP’s protective effect is that even short-term application can lead to significant biological effects due to its conformational stability when bound to membranes and its immediate, but long-lasting effects on intracellular signaling (Vaudry et al. 1998; Krishnadas et al. 2003). Various studies have shown that PACAP administration is sufficient to exert neuroprotective effects in various rodent models (Nonaka et al. 2012; Cherait et al. 2021). However, as suggested by some specialists, a more stable analogs with enhanced bioavailability could be helpful for PACAP use as treatment for neuronal diseases. For this purpose, many structural modifications have been employed to enhance PACAP stability and bioavailability in the brain, reduce its metabolic degradation, and then extend its half-life, improving by the way its pharmacokinetics and pharmacodynamics properties. This is the case for example of the PACAP glycopeptide analogs, 2LS80Mel and 2LS98Lac, which exert potent neuroprotective effects and exhibit anti-inflammatory activity in animal models of TBI and in a toxin lesion model of Parkinson’s disease (Apostol et al. 2022); of the Ac-[Phe(pI)6, Nle17]PACAP(1–27), which exerts a strong neuroprotection without significantly affecting the heart rate (Lamine et al. 2016); or of the acetyl-[Ala15, Ala20]PACAP38-propylamide (Bourgault et al. 2008; 2009a), with enhanced serum stability but which seems to behave differently from PACAP in some experiments (Ladjimi et al. 2019; 2020). If PACAP stabilized derivatives, which retain all their biological activity, are an interesting option for the development of a therapeutic agent, it must be kept in mind that the acetylation, replacement of original amino acids by alanine in position 15 and 20, or the addition of a propylamide group block peptidase activity; they may also induce an activity bias that will not always be easy to characterize well.

In parallel to the creation of peptide analogs, the search for small molecules acting on PACAP receptors is also of interest for the pharmaceutical industry. These molecules are often cheaper to produce and can be more stable. In general, this leads to the discovery of PACAP antagonists because of the two-step binding model of activation of PACAP (Takasaki et al. 2018; Lu et al. 2022; Xu et al. 2024). However, Omeros Corporation has succeeded in identifying small molecules capable of interacting with class B G-protein coupled receptors (GPCR) and inducing their stimulation (Omeros 2013). These molecules would be allosteric modulators that interact in a different way than classical activators of class B GPCR. Recent advances have led to the identification of non-peptide small-molecule antagonists of the PACAP specific receptor that have shown efficiency in penetrating the BBB and reducing some PACAP undesirable effects (Takasaki et al. 2018; Shintani et al. 2022). It is worth noting that the search for PACAP agonists and antagonists is a very active area of research for now.

A third solution, as proposed for the BBB uptake, would be to use delivery methods that prevent PACAP degradation, such as nano-delivery systems or adenovirus vectors that will release PACAP only in the target brain area. The advantage of adenovirus vectors is that they allow both a localized and continuous release of PACAP. However, the absence of studies regarding the effects of a persistent exposure to PACAP could be a drawback.

Alternatively to the administration of agonists, the increase of endogenous PACAP levels through the use of a cocktail of peptidase inhibitors could be promising. One of the advantages of such a strategy is that some of these molecules, such as linagliptin, are already on the market (Aljohani et al. 2024), which will facilitate regulatory procedures and make their use in the clinic easier. Furthermore, the fact that recent publications show that linagliptin exerts neuroprotective effects (Wiciński et al. 2019; Abhangi and Patel 2022; Arab et al. 2023) should encourage us to explore this avenue.

### Action via Various Receptors and Wide Distribution

**Fig. 4:**
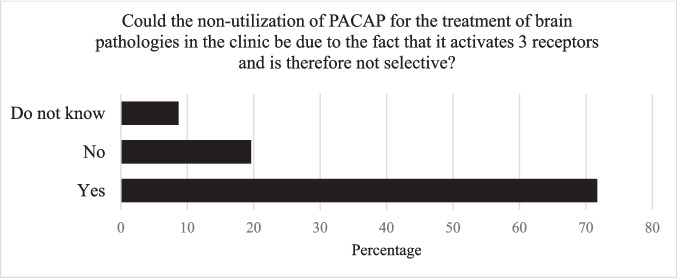
Specialists surveyed response regarding the three PACAP receptors’ activation

The side effects on peripheral tissues are perceived as an obstacle to the development of a treatment targeting the brain by three-quarters of the specialists surveyed (Fig. 4). However, it is important to keep in mind that PACAP acts through three different GPCRs called PAC1, VPAC1, and VPAC2 (Kumar et al. 2011). PAC1 has high affinity for PACAP and low affinity for vasoactive intestinal polypeptide (VIP), whereas VPAC1 and VPAC2 have similar affinity for PACAP and VIP (Suda et al. 1992; Vaudry et al. 2000; Gabriel et al. 2019). In the brain, PAC1 is more expressed than VPAC1 or VPAC2 (Jolivel et al. 2009), whereas VPAC1 and VPAC2 are abundant in various peripheral organs such as the lung and thymus (Vaudry et al. 2000).

It is well known that PACAP peripheral side effects occur mainly through VPAC1 and VPAC2 receptors because these two receptors are predominantly expressed in peripheral tissues (Leceta et al. 2000; Fizanne et al. 2004; Moody and Jensen 2016; Ago et al. 2021; Bourgault et al. 2009b). In particular, VPAC2 is associated with some cancer development, hypotension, water retention, tachycardia, etc. (Bourgault et al. 2009b; Ago et al. 2021; 2023). Furthermore, at the brain level, the activation of the VPAC2 by an IV administration of its specific agonist, Bay 55–9837, after transient MCAO (tMCAO), increases stroke severity in rats (Darsalia et al. 2013). In fact, the neuroprotective effects of PACAP are mostly driven by the PAC1 receptor, which promotes neural survival and synaptic plasticity (Vaudry et al. 2009). VPAC1 would also exert a slight neuroprotective action via its capacity to modulate the immune response. Therefore, researchers have sought to develop analogs that can specifically target certain receptors. This is the case of the Ac-[Phe(pI)6, Nle17]PACAP(1–27) which exerts enhanced affinity for PAC1 compared to VPAC2 and therefore displays potent neuroprotective activity and reduced in vivo cardiovascular side effects in a Parkinson’s disease model (Lamine et al. 2016).

**Fig. 5:**
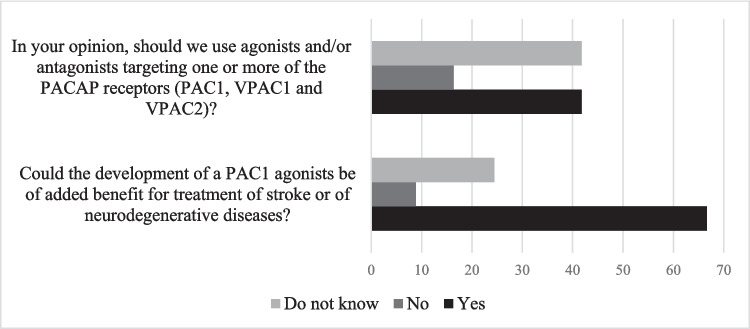
Specialists’ answers regarding the interest to develop PACAP analogs

Two-thirds of the specialists interviewed in our survey consider that targeting the PAC1 receptor via the development of specific agonists is a promising strategy for treating brain disorders (Fig. 5). However, the activation of the PAC1 receptor can also exacerbate certain diseases. In fact, each receptor activates various transduction pathways such as cAMP/PKA, phospholipase C (PLC), ERK, intracellular calcium, protein kinase C (PKC), phosphoinositide 3-kinase (PI3 K/Akt), and phospholipase D (PLD), among others. The signaling cascades activated by PACAP depend from one cell to another and can even change depending on the state of the cell (Nicot and DiCicco-Bloom 2001). This is related to the fact that not all cells express the same intracellular machinery, but also that there are several splice variants of the PAC1 receptor, each with different couplings to small G proteins (Gabriel et al. 2019). The expression of these splice variants can be different between an immature neuron and a mature one, leading sometimes to opposite effects (Nicot and DiCicco-Bloom 2001). PACAP receptor expression can also be modified in some brain pathologies (Rivnyak et al. 2018; Feher et al. 2023). By consequence, the development of analogs (agonist or antagonist) or even allosteric modulators that target a specific PACAP receptor variant depending on the brain pathology could be a useful strategy to reduce PACAP undesirable side effects and enhance its efficiency. However, in view of the large number of PAC1 receptor isoforms (Lu et al. 2022), each of which has its own specificity of coupling, their implication in the pathogenesis of brain diseases must be elucidated, and their specificity of binding to PACAP has to be refined to determine whether discriminating analogs can be developed. Depending on the results that will be obtained, it may be necessary to combine simultaneously or successively several molecules, such as a PAC1 and/or VPAC1 agonist and a VPAC2 antagonist. This could be the case for the treatment of diseases with multifactorial components, such as multiple sclerosis, where the current therapeutic agents are able to reduce the severity of the immune response at an early stage but fail to protect the neurodegenerative aspect of the disease (Tan and Waschek 2011). In the present case, targeting the VPAC1 receptor in the early stages of multiple sclerosis could help to modulate the immune response, while activating the PAC1 receptor at later stages would promote neuroprotection, as mentioned by some of the experts interviewed.

Based on structure–activity relationship analysis, a deletion of the N-terminal residues in a PACAP derivative molecule can generate a peptide’s receptor antagonist, as seen with DPP-IV (Green et al. 2006; Bourgault et al. 2008). Conversely, subtle modifications of the peptide play a role in VPAC receptor selectivity (Lu et al. 2022), as in the case of PG 99–465, an N-terminal myristoylated and C-terminal elongated VIP analog (Moreno et al. 2000). Other strategies could be employed to design selective therapeutic analogs targeting PACAP receptors, as discussed by Lu et al. (2022). The Table 1 resume some PAC1 analogs based on these peptide engineering approaches.

**Table 1:** Amino acid sequence of PACAP and some PAC1 analogs

PACAP/analogs	Sequences	Receptors	References
PACAP38	HSDGIFTDSYSRYRKQMAVKKYLAAVLGKRYKQRVKNK	PAC1 VPAC1/2 GPR55 MRG B2/B3/X2	Vaudry et al. 2009; Alhouayek et al. 2018; Pedersen et al. 2019; Foster et al. 2019; Sbei et al. 2023
PACAP27	HSDGIFTDSYSRYRKQMAVKKYLAAVL	PAC1 VPAC1/2 GPR55?	Vaudry et al. 2009; Alhouayek et al. 2018; Foster et al. 2019
PACAP23	HSDGIFTDSYSRYRKQMAVKKYL	PAC1	Lamine et al. 2019
[Iaa1]PACAP38	Iaa-SDGIFTDSYSRYRKQMAVKKYLAAVLGKRYKQRVKNK	PAC1	Ramos-Álvarez et al. 2015
[Iac1]PACAP38	Iac-SDGIFTDSYSRYRKQMAVKKYLAAVLGKRYKQRVKNK	PAC1/VPAC1	Ramos-Álvarez et al. 2015
Acetyl-[Ala15, Ala20] PACAP-38-propylamide	Ac-HSDGIFTDSYSRYR-Ala-QMAV-Ala-KYLAAVLGKRYKQRVKNK-PA	PAC1	Bourgault et al. 2009aª
[Ala7]PACAP27	HSDGIF-Ala-DSYSRYRKQMAVKKYLAAVL	PAC1/VPAC1	Bourgault et al. 2009aª
[Trp6 Nle17]PACAP27	HSDGI-Trp-TDSYSRYRKQ-Nle-AVKKYLAAVL	PAC1/VPAC1	Lee-Gosseline 2017
Ac-[Phe(pI)(6), Nle(17)]PACAP(1–27)	Ac-HSDGI-Phe(pI)-TDSYSRYRK-Nle-MAVKKYLAAVL	PAC1/VPAC1/2	Lamine et al. 2016

So far, it has been easier to develop PAC1 antagonists, such as the PA-8 (Takasaki et al. 2019) than specific agonists because antagonists only need to bind the receptor, while agonists need to both bind and activate the receptor, which seems to occur at two distinct places (Bourgault et al. 2011; Dejda et al. 2011a). We can nevertheless think that the integration of artificial intelligence (AI)-driven design together with molecular dynamics simulations will soon lead to the identification of promising PACAP analogs. Indeed, drug discovery and development are currently undergoing revolutionary changes, from traditional methods to AI and machine learning (ML) technologies that contribute to faster and more efficient discoveries (Lappala 2024). The use of AI algorithms, such as evolutionary algorithms and neural networks, streamlines the identification of potential drug candidates by analyzing vast datasets and predicting molecular interactions (Elend et al. 2022) and has been able to generate structurally diverse PROTACs with superior binding affinities, quantitatively evaluating binding energies, and aligning computational predictions with experimental data, thus improving drug design efficacy and reliability (Chou et al. 2024). Molecular dynamics simulations through the integration of quantum chemistry provide insights into the atomic-level interactions between drugs and target proteins, enhancing the precision of predictions regarding molecular interactions and toxicity risks (Pasupuleti 2024).

Although there is a consensus that specific PACAP receptors analogs could overcome its pharmacokinetics limitations, experts consider that the impact of a long-term activation of the PACAPergic system needs to be addressed. We also need to better understand the potential of homo- and hetero-dimerization, the complex signaling cascades, and the neuroprotective contribution of every PACAP receptors variants in various cellular contexts.

### Involvement in Numerous Disorders

There is no consensus among the experts whether the peripheral side effects, such as cardiac issues, prevent the use of PACAP for brain neuroprotection, even if 38% of people interviewed consider that the ability of the peptide to promote migraine precludes its use as a neuroprotectant in humans (Table 2). In fact, many experts do not take a position on this aspect, probably because it is very complex, since PACAP acts in at least 40 distinct pathological conditions, in which it exerts either protective or deleterious effects (Denes et al. 2019). We can consider that the effects of PACAP on the cardiovascular system (Birk et al. 2007; Seeliger et al. 2010; Sarszegi et al. 2019; Amin et al. 2014), feeding behavior (Nakamachi et al. 2019; Maunze and Choi 2023), migraine (Schytz et al. 2009; Edvinsson et al. 2018; Ghanizada et al. 2020), cancer (Valdehita et al. 2007, 2009; Langer et al. 2022), and neuropsychiatric disorders (Hashimoto et al. 2007, 2010; Ressler et al. 2011; Lohoff et al. 2008; Vacic et al. 2011; Gozes 2016; Donahue 2016) are issues that will need to be monitored before use in clinic.

**Table 2:** Specialists’ answers regarding PACAP disorder’s involvement

Question statement	Yes	No	Do not know	Maybe/not really
**In your opinion, is the clinical use of PACAP limited by its involvement in the pathogenesis of many disorders?**	27%	34%	36%	3%
**PACAP has been known to cause some peripheral side effects, such as cardiac issues. In your opinion could this be why it has not been used as a neuroprotectant in humans so far?**	27%	27%	41%	5%
**Could the effects of PACAP on cerebral hemodynamics and/or its ability to promote migraine preclude its use as a neuroprotectant in humans?**	38%	22%	38%	2%

Nevertheless, the situation is difficult to address since all the mechanisms by which PACAP acts are not well elucidated and could depend on genetic factors. Indeed, in cancers, a modification of the receptor density is observed (Reubi et al. 2000; Moody and Jensen 2016), whereas in neuropsychiatric diseases (schizophrenia, autism spectrum disorder, etc.), an overexpression of VPAC2 receptors is a risk factor (Vacic et al. 2011; Li et al. 2016; Ago et al. 2023). Another example concerns the pathophysiology of migraines, which was initially considered to involve the PAC1/cAMP pathway (Ashina 2020). However, recent findings suggest that the situation is intricate since VPAC1 and VPAC2 receptors might also be implicated (Pellesi et al. 2020). This hypothesis is reinforced by the failure of AMG301, a PAC1 receptor monoclonal antibody acting as an antagonist, to prevent migraine attacks (Ashina et al. 2021). This clinical trial (ClinicalTrials.gov: NCT03238781) questions the mechanism by which PACAP induces migraine and suggests that it could be mediated by a specific splice variant or even an undetermined PACAP signaling pathway (unknown receptor) independent of PACAP traditional receptors (Kuburas and Russo 2023). This might involve, for example, the orphan Mas-related G-protein coupled receptor MRG B2/B3/X2 reported to mediate PACAP-induced mast cell degranulation (Pedersen et al. 2019; Sbei et al. 2023), known to play a role in migraine pathogenesis (Levy et al. 2007). However, other candidates exist such as the GPR55, known to be activated by PACAP fragments and to contribute to migraine and other disorders such as cancer or pain (Alhouayek et al. 2018; Foster et al. 2019). And de facto, a clinical study (ClinicalTrials.gov number, NCT05133323) with the monoclonal antibody Lu AG09222, targeting PACAP rather than its receptor, is close to clinical use in migraine patients (Ashina et al. 2024). This illustrates that it is important to take into account the consequences of stopping various PACAP signaling pathways, but systemic blocking of the PAC1 receptor has to be handled carefully, as it will also abolish the potential protective effects of the endogenous PACAPergic system (Ashina et al. 2024).

In any case, these examples illustrate the necessity to further investigate the mechanisms responsible for the effects of PACAP in the development of various diseases before considering its clinical use. In the current state of knowledge, PACAP specialists recommend for clinical applications the use of low doses, a local targeted delivery route, and the clinical management of any incidence. Some of the specialists believe that the adverse effects could be problematic in individuals with pre-existing issues such as cardiovascular disease or a propensity for migraines but point out that globally the benefit/cost should tilt in favor of the clinical use of PACAP, since side effects can be managed through the use of other drugs concomitantly with PACAP neuroprotective treatment. This was illustrated with sumatriptan, which has been shown effective in treating PACAP-induced migraine attacks (Amin et al. 2014). The use of PACAP analogs, such as the Ac-[Phe(pI)(6), Nle(17)]PACAP(1–27), may also offer a viable opportunity to bypass some of its potential side effects as it exerts a potent neuroprotective activity with fewer cardiovascular side effects compared to the native peptide (Lamine et al. 2016). As proposed by experts, the use of a partial agonist could also help to diminish PACAP drawback effects. This is the case with PACAP23, a short amidated PAC1 agonist, which exerts a strong neuroprotective effect in Parkinson’s disease models, similar to the native PACAP, but with a reduced affinity to the receptor (Lamine et al. 2019), which means that it will probably have fewer side effects.

### Delivery Routes and Posology Issues

Forty percent of the specialists consider that PACAP administration delivery route problems may have precluded its use for the treatment of brain diseases in clinic (Table 3), while 38% of the specialists believe that it may not be an obstacle. When an IV administration of PACAP is considered for the treatment of neurodegenerative diseases, 21% of the specialists surveyed consider that it should be performed in a continuous manner to maintain a stable PACAP blood level and avoid the occurrence of peak levels, thus limiting the risk of side effects due to bolus administrations. Conversely, 7% of respondents recommend the use of a bolus administration once to avoid desensitization of PACAP receptors considering that it will be sufficient to obtain a PACAP neuroprotective effect, while 22% are in favor of repeated injections every hour or a couple of hours to sustainably maintain the transduction pathways activated by PACAP. On top of that, a quarter of the specialists consider that another delivery route would be more efficient than the IV one and should be chosen depending on the given disease, its neurodegenerative state, and the rate of tissue decay. Half of PACAP specialists consider that the intranasal (IN) administration of PACAP for the treatment of neurological disorders could be the alternative treatment route even though 27% of the interrogated experts consider that it may cause a nasal epithelium irritation, which would hinder prolonged use. Even if the intranasal route is considered promising following animal trials, it remains to be confirmed for 51% of the specialists that the absorption of PACAP through the nasal epithelium in humans is sufficient for clinical application.

**Table 3:**
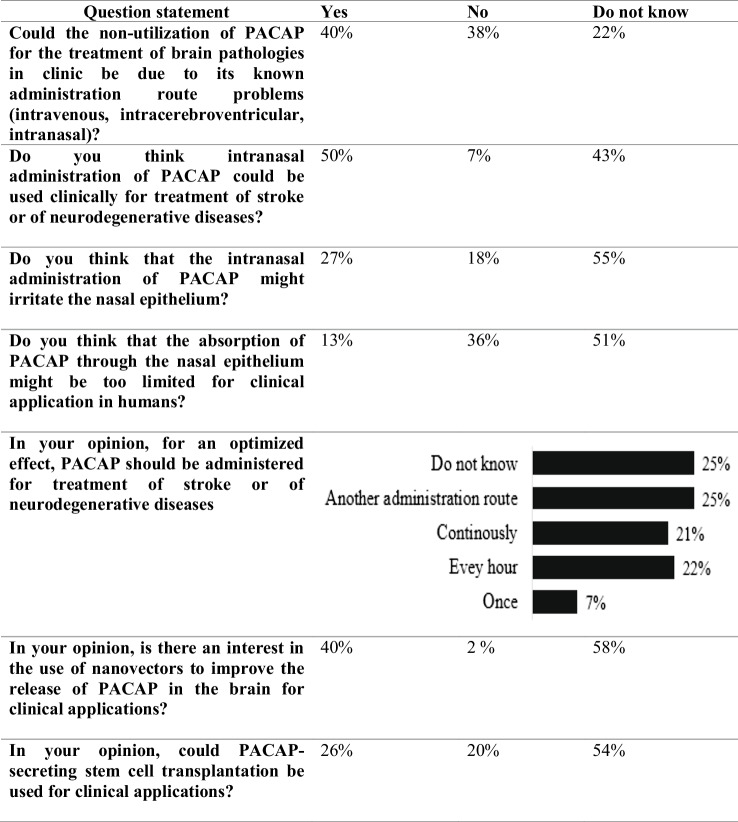
Response of the survey regarding PACAP unknown delivery routes and posology

**Fig. 6:**
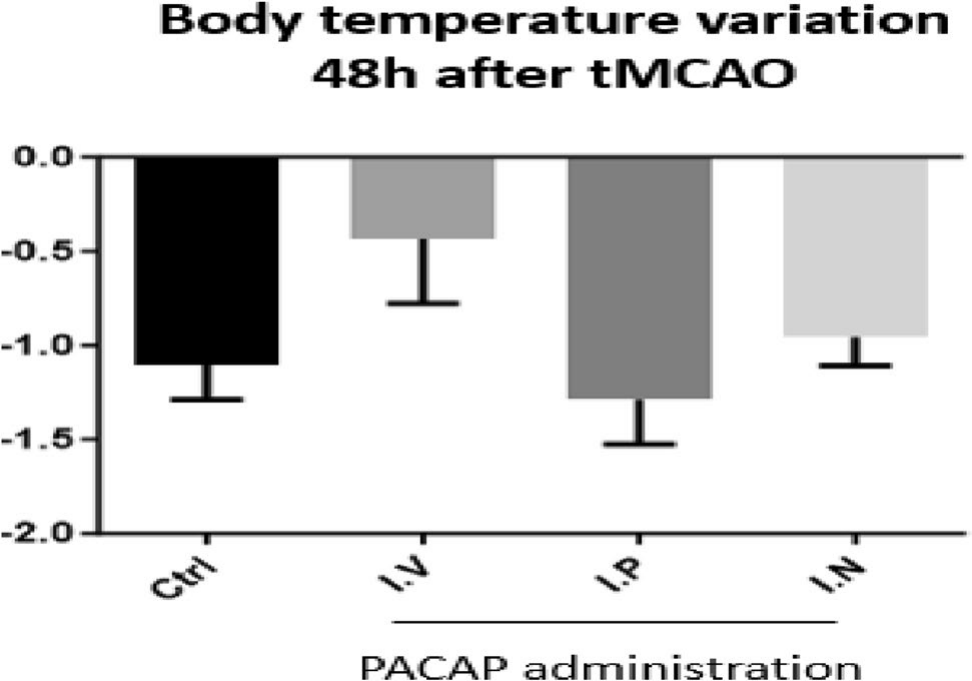
Body temperature variations after PACAP administration intranasally (10 µg in 10 µl; IN;* n* = 7), intravenously (200 μl at a concentration of 0.02 μg/kg with 100 μl in the form of a bolus and then 100 μl by infusion over a 30-min period; IV; *n* = 7), or intraperitoneally (200 μl in the form of a bolus at a concentration of 0.02 μg/kg; IP; *n* = 8) in tMCAO mice. The control group represents mice with tMCAO treated 10 min after the reperfusion by intranasal administration of NaCl (0.9%; Ctrl; *n* = 7)

Several administration routes for PACAP delivery have been tested. In clinical studies, the privileged administration route of PACAP has been the systemic one, mostly IV (Li et al. 2007; Birk et al. 2007; Amin et al. 2012; Amin et al. 2014; Ghanizada et al. 2020). In preclinical studies, the IV delivery of PACAP exerts a neuroprotective action (Dejda et al. 2011b; Deguil et al. 2010; Lamine et al. 2016; Cherait et al. 2021). This administration pathway allows a delivery of the peptide to the rat brain with a fast uptake in the hypothalamus and hippocampus and with a slower assimilation observed in aged animals (Nonaka et al. 2002). Alternatively, PACAP has also been administered intraperitoneally (IP), subcutaneously, and orally (Reglodi et al. 2018a). A systemic delivery of PACAP could compromise its therapeutic applications as an efficient neuroprotective drug due to its rapid plasmatic degradation and wide range of undesirable effects. In human blood, the half-life of PACAP38 ranges between 5 and 10 min (Li et al. 2007) after an IV infusion to rats. Furthermore, as already mentioned, a systemic application of PACAP may affect other organs that are not the target of the treatment. Most neuroprotective effects of PACAP have been reported on in vitro cultured cell models, a situation which ignores the numerous interactions of the PACAP/PAC1 system with its global environment at the body level (Reglodi et al. 2018b). To overcome this problem, PACAP topical administration has been proven effective in several pathological conditions such as in eye diseases. As the retina is an extended part of the central nervous system, it is worth noting that a promising route of administration is in the form of eye drops not only in corneal but also in retinal diseases. In rodent experiments, PACAP has been shown to pass through the ocular barriers and reach the retina in concentrations high enough to exert neuroprotection in models of different retinal diseases, like glaucoma and ischemia (Werling et al. 2016; Patko et al. 2023). A topical injection of the peptide into the brain or cerebrospinal fluid is also effective but is not suitable for clinical use because of its invasiveness (Brown and Liu 2014). In addition, intrathecal delivery may induce some adverse effects in peripheral organs due to a potential systemic distribution of the peptide (Farnham et al. 2011; Calias et al. 2014). Thus, the optimal delivery route of PACAP for neuroprotective therapies could be the nose-to-brain one, which has been proposed by several studies (Rat et al. 2011; Guo et al. 2021; Cherait et al. 2021). This non-invasive easy route offers a rapid delivery to the brain and circumvents the systemic distribution of the peptide and thus its peripheral side effects (Cherait et al. 2023). The efficiency of PACAP IN delivery to the brain is around 1% to 5% of the total administered peptide (Dhuria et al. 2010) and leads to a direct transfer of the compound to the brain via the olfactory/trigeminal pathways, in similar or higher concentrations than the amount obtained by systemic administration (Hoekman and Ho 2011; Yang et al. 2013) since it can increase the peptide concentration by 100-fold in various brain areas (Dhuria et al. 2010). In a preclinical study, the IN delivery of PACAP in a tMCAO stroke model is much more efficient than the IV or IP administration (Cherait et al. 2021). Furthermore, IN delivery does not affect food intake or body weight (Cherait et al. 2021). In the same way, as shown in Fig. 6, it has no influence on body temperature (unpublished work).

However, experimental results obtained in rodents are not readily transferable to humans due to some anatomical and physiological differences and may fail in clinical tests due to this translational problem, as pointed out by 20% of the interrogated specialists (Table 4). Therefore, clinical trials are needed to assess the potential applications of PACAP and PACAP-related molecules for the treatment of neurological disorders and confirm their safety. Once this is done, some issues need to be improved, such as the optimum PACAP formulation and delivery approach. To assess the quantity of PACAP that reaches the olfactory region of various formulations for IN delivery, the use of a 3D printed nasal casts (Rigaut et al. 2023) could be useful. The experts interviewed particularly believe in the IN administration route of PACAP for the treatment of neurological disorders while limiting side effects. As approved by 40% of the experts interviewed (see Table 3), this could involve nanosized drug delivery carriers such as the polymer- and lipid-based nanocarriers with biorecognitive ligands and permeation enhancers to improve the direct transport of the peptide from the nasal cavity to the brain area of interest or from the bloodstream to the central nervous system (Samaridou and Alonso 2018; Borrajo and Alonso 2022). The use of stem cells has also been proposed by one quarter of the specialists. However, the use of stem cells secreting PACAP (Brifault et al. 2015) needs further research to fully understand its potential applications, safety, and limitations in clinic.

**Table 4:** Response of the survey regarding PACAP’s other drawbacks for its use in clinic

Question statement	Yes	No	Do not know
**Could the non-utilization of PACAP for the treatment of brain pathologies in the clinic stem from the fact that the neuroprotective effects of PACAP reported in animals and cell cultures models were not achieved in humans?**	20%	27%	53%
**Could the non-utilization of PACAP for the treatment of brain pathologies in the clinic stem from its transient effects (anti-inflammatory, anti-apoptotic, etc.)?**	13%	38%	49%
**Do you think that the patents already filed regarding PACAP applications for treatment of stroke or of neurodegenerative diseases are limiting the development of clinical use by the pharmaceutical industry?**	9%	16%	75%

## Other Drawbacks

Some patents already exist regarding the applications of PACAP and analogs for treatment of stroke or of neurodegenerative diseases such as the EP3712164 A1, EP2161282, and EP1098906. However, less than 10% of the specialists interviewed consider that this is a limiting factor for the development of a clinical use by the pharmaceutical industry (Table 4). We can nevertheless note that 3/4 of the people questioned have no opinion on this point, which shows that the impact of patents in the development of drugs is not always well understood by academic researchers. Companies interested in the intranasal administration of PACAP for therapeutic applications seem to count on the development of new analogues to bypass existing patents.

The PACAP transient anti-inflammatory or anti-apoptotic effects do not seem to be considered a major obstacle for the treatment of brain pathologies (Table 4). This is probably due to the fact that many preclinical studies have already demonstrated the strong neuroprotective effect of PACAP and the mechanisms involved (Djeda and al. 2011; Nonaka and al. 2012; Cabezas-Llobet and al. 2018; Cherait and al. 2021).

## Conclusion

PACAP is involved in pleiotropic neurological functions that make it an attractive target for the treatment of various brain disorders, as shown by numerous experimental studies (Cherait and al. 2021; 2023). However, although there is now enough evidence to encourage PACAP clinical trials for the treatment of neurological disorders, there is still strong reticence due to its poor pharmacokinetics properties and wide range of potential side effects. Yet, PACAP’s potential side effects could be overturned via the use of low doses of PACAP, an efficient administration route, and the determination of the optimal formulation. Among the other possibilities to overcome PACAP’s drawbacks, such as its pharmacokinetics limitations, there is the use of PACAP analogs or even non-peptidic agonistic or antagonistic molecules. Such developments are time- and cost-consuming, which could explain a decline in the interest in PACAP in favor of other drug candidate molecules that are easier to bring to the market. Nevertheless, taking into account all the PACAP beneficial effects compared to its potential side activities and the urgent need for an efficient treatment of various neurological disorders, the assessment of the cost–benefit ratio of PACAP needs to be clarified as soon as possible via some clinical trials to establish its effectiveness and safety in humans. We can believe that the recent regain of interest for the design of PACAP analogs, together with the arrival of new digital tools, will bring very useful tools to move toward clinical studies.

## Supplementary Information

Below is the link to the electronic supplementary material.

Supplementary file1 (DOCX 17 KB)

## Data Availability

Data are available from the corresponding author on request.
