# Supplementary material for: More Than Three Decades After Discovery of the Neuroprotective Effect of PACAP, What Is Still Preventing Its Clinical Use?
Source: J Mol Neurosci. 2025 Jun 21;75(3):80. doi: 10.1007/s12031-025-02366-z (PMC12182478; doi:10.1007/s12031-025-02366-z)
Supplement: Supplementary file 1 — Supplementary file1 (DOCX 17 KB) [file 12031_2025_2366_MOESM1_ESM.docx]

Survey Questionary

Despite the powerful neuroprotective effects that have been studied for more than 20 years now, why do you think PACAP is still not used in clinic for treatment of cerebral ischemia or some neurodegenerative diseases?

This survey aims at figuring out where we are regarding the clinical use of PACAP and what is still preventing its use for the treatment of cerebral ischemia or some neurodegenerative diseases. We thank you in advance for the time spent answering these 21 questions. Do not hesitate to mention some references which support your answers.

Q1. Do you plan to conduct a clinical study with PACAP or PACAP related molecules for the treatment of stroke and/or of neurodegenerative diseases?

Yes No

If yes, could you give some information regarding the study and the pathology you would like to treat with PACAP? Why this pathology?

|  |
| --- |

Q2. Are you aware of clinical studies with PACAP or PACAP related molecules for the treatment of stroke and/or of neurodegenerative diseases which should start soon?

Yes No

If yes, could you give some information regarding the studies that will be conducted?

|  |
| --- |

Q3. PACAP has been known to cause some peripheral side effects, such as cardiac issues. In your opinion could this be why it has not been used as a neuroprotectant in humans so far?

Yes No Do not know

If yes, could you expatiate on, the main side effects which prevent the use of PACAP in clinic? Do you see a method to bypass them?

|  |
| --- |

Q4. Could the effects of PACAP on cerebral hemodynamics and/or its ability to promote migraine preclude its use as a neuroprotectant in humans?

Yes No Do not know

If yes, do you see a way of bypassing this problem? If no, could you explain your answer?

|  |
| --- |

Q5. Could the non-utilization of PACAP for the treatment of brain pathologies in the clinic be due to its known administration route problems (intravenous, intracerebroventricular, intranasal)?

Yes No Do not know

If yes, do you see a way of bypassing the problem? If no, could you explain your answer?

|  |
| --- |

Q6. Could the non-utilization of PACAP for the treatment of brain pathologies in the clinic be due to its metabolic instability?

Yes No Do not know

If yes, do you think using PACAP related molecules could bypass this problem? Or could you think of another solution? If no, could you explain your answer?

|  |
| --- |

Q7. Could the non-utilization of PACAP for the treatment of brain pathologies in the clinic stem from its low bioavailability?

Yes No Do not know

If yes, do you think using PACAP related molecules could bypass this problem? Or do you think of another solution? If no, could you explain your answer?

|  |
| --- |

Q8. Could the non-utilization of PACAP for the treatment of brain pathologies in the clinic be due to the fact that it activates 3 receptors and is therefore not selective?

Yes No Do not know

If yes, do you think that the development of selective agonists will solve this problem? Or could you think of another solution? If no, could you explain your answer?

|  |
| --- |

Q9. Could the non-utilization of PACAP for the treatment of brain pathologies in the clinic stem from the fact that the neuroprotective effects of PACAP reported in animals and cell cultures models were not achieved in humans?

Yes No Do not know

If yes, could you indicate the groups or compagnies which carried out these studies? If no, could you explain your answer?

|  |
| --- |

Q10. Could the non-utilization of PACAP for the treatment of brain pathologies in the clinic stem from its transient effects (anti-inflammatory, anti-apoptotic, etc)?

Yes No Do not know

If yes, do you see some applications where a transient neuroprotective effect of PACAP would be of interest? If no, do you think the effects of PACAP last long enough?

Q11. In your opinion, is the clinical use of PACAP limited by its involvement in the pathogenesis of many disorders?

Yes No Do not know

If yes, what are, according to you, the main solutions to bypass this problem for the use of PACAP in clinic? If no, could you explain your answer?

|  |
| --- |

Q12. Could the development of a PAC1 agonist be of added benefit for treatment of stroke or of neurodegenerative diseases?

Yes No Do not know

If yes, which analog would you think would be most suitable for clinical use? If no, could you explain your answer? Would it be more interesting to have a molecule which targets 2 or 3 of the receptors rather than only one?

|  |
| --- |

Q13. Do you think intranasal administration of PACAP could be used clinically for treatment of stroke or of neurodegenerative diseases?

Yes No Do not know

Q14. Do you think that the intranasal administration of PACAP might irritate the nasal epithelium?

Yes No Do not know

Q15. Do you think that the absorption of PACAP through the nasal epithelium might be too limited for clinical application in humans?

Yes No Do not know

Do you have some comments regarding the potential and limits of PACAP intranasal administration?

|  |
| --- |

Q16. In your opinion, for an optimized effect, PACAP should be administered for treatment of stroke or of neurodegenerative diseases: Continuously As a bolus, once As a bolus every couple of hours In another way

Please give arguments explaining your answer.

|  |
| --- |

Q17. In your opinion, should we use agonist and/or antagonists targeting one or more of the PACAP receptors (PAC1, VPAC1 and VPAC2)?

Yes No Do not know

If yes, what could the optimal temporal combination for the administration of PAC1, VPAC1 and/or VPAC2 agonist or antagonists for treatment of stroke or of some neurodegenerative diseases be? If it is disease-specific, please explain why? If no, could you argue your answer?

|  |
| --- |

Q18. In your opinion, is there an interest in the use of nanovectors to improve the release of PACAP in the brain for clinical applications?

Yes No Do not know

If yes, what type of vectors and why? If no, could you explain your answer?

|  |
| --- |

Q19. In your opinion, could PACAP-secreting stem cell transplantation be used for clinical applications?

Yes No Do not know

If yes, what type of cells would you recommend using? If no, could you explain your answer?

|  |
| --- |

Q20. Do you think that the patents already filed regarding PACAP applications for treatment of stroke or of neurodegenerative diseases are limiting the development of clinical use by the pharmaceutical industry?

Yes No Do not know

If yes, do you see a way to bypass the problem? If no, could you explain your answer?

Q21. Do you see other possible reasons why PACAP has not been used in clinic for treatment of stroke and/or of neurodegenerative diseases so far?

|  |
| --- |
